# Assessment of trade-offs between feed efficiency, growth-related traits, and immune activity in experimental lines of layer chickens

**DOI:** 10.1186/s12711-021-00636-z

**Published:** 2021-05-06

**Authors:** Tatiana Zerjal, Sonja Härtle, David Gourichon, Vanaïque Guillory, Nicolas Bruneau, Denis Laloë, Marie-Hélène Pinard-van der Laan, Sascha Trapp, Bertrand Bed’hom, Pascale Quéré

**Affiliations:** 1 INRAE, AgroParisTech, Université Paris-Saclay, GABI, 78350 Jouy-en-Josas, France; 2 Avian Immunology Group, Department for Veterinary Sciences, LMU Munich, Munich, Germany; 3 INRAE, PEAT, 37380 Nouzilly, France; 4 INRAE, UMR 1282, ISP, Université de Tours, 37380 Nouzilly, France; 5 Present Address: ISYEB, Muséum National D’Histoire Naturelle, CNRS, Sorbonne Université, EPHE, Université Des Antilles, 75005 Paris, France

## Abstract

**Background:**

In all organisms, life-history traits are constrained by trade-offs, which may represent physiological limitations or be related to energy resource management. To detect trade-offs within a population, one promising approach is the use of artificial selection, because intensive selection on one trait can induce unplanned changes in others. In chickens, the breeding industry has achieved remarkable genetic progress in production and feed efficiency over the last 60 years. However, this may have been accomplished at the expense of other important biological functions, such as immunity. In the present study, we used three experimental lines of layer chicken—two that have been divergently selected for feed efficiency and one that has been selected for increased antibody response to inactivated Newcastle disease virus (ND3)—to explore the impact of improved feed efficiency on animals’ immunocompetence and, vice versa, the impact of improved antibody response on animals’ growth and feed efficiency.

**Results:**

There were detectable differences between the low (R+) and high (R−) feed-efficiency lines with respect to vaccine-specific antibody responses and counts of monocytes, heterophils, and/or T cell population. The ND3 line presented reduced body weight and feed intake compared to the control line. ND3 chickens also demonstrated an improved antibody response against a set of commercial viral vaccines, but lower blood leucocyte counts.

**Conclusions:**

This study demonstrates the value of using experimental chicken lines that are divergently selected for RFI or for a high antibody production, to investigate the modulation of immune parameters in relation to growth and feed efficiency. Our results provide further evidence that long-term selection for the improvement of one trait may have consequences on other important biological functions. Hence, strategies to ensure optimal trade-offs among competing functions will ultimately be required in multi-trait selection programs in livestock.

**Supplementary Information:**

The online version contains supplementary material available at 10.1186/s12711-021-00636-z.

## Background

All organisms are inherently constrained by the resources that are available to them, and must therefore allocate these limited resources among competing functions [1–3]. This idea is central to the biological concept of trade-offs, which represent the costs incurred when a change in one trait results in an unfavorable change in another [4], thus establishing a limit for the total fitness/function that is achievable. The manifestation of trade-offs is often identified as negative phenotypic and/or genetic correlations among traits, although exceptions have been reported [5, 6]. The processes mediating trade-offs are complex and can involve both energetic and non-energetic mechanisms [7]. In the last two decades, studies of the physiological bases of life history trade-offs have highlighted the importance of hormonal control of antagonistic traits [7, 8] and of oxidative stress [9], which have both been reported to regulate certain trade-offs.

A promising means for detecting within-population trade-offs is the use of artificial selection, because intensive selection on one trait can induce unplanned changes in others. In the domestic chicken, strong selection for intensive, efficient, and specialized production has favored productiveness over other physiological processes. Indeed, over the last 60 years, the commercial poultry breeding industry has achieved remarkable genetic progress for economic criteria such as growth rate, meat or egg production, and feed efficiency. For example, Zuidhof et al. [10] reported that, compared to a broiler line from the 1950s, modern broilers have a growth rate that is more than 400% faster, with a concomitant 50% reduction in feed conversion ratio (estimated as the ratio between the mass of feed consumed and the total weight gain for a particular period). Similarly, by revisiting data from more than five decades of performance testing of layer chickens in North Carolina, Anderson et al. [11] described a steady increase in egg production for both white and brown egg-laying strains, with an improvement rate of approximately 0.5 egg per year, a reduction in body weight of about 30%, and a feed conversion ratio (expressed as kg of feed per kg of eggs produced) approaching 2.0. However, this remarkable progress seems to have had consequences for other major physiological traits. Field observations suggest that commercial chickens generally display weaker immune capacities, as indicated by an increased susceptibility to infectious diseases and reduced adaptive immune responsiveness [12–14]. This undesirable phenomenon might be explained by the resource allocation theory of Beilhartz [15], from which we can expect that when an animal is genetically driven towards high production and efficiency, fewer resources will be left for other life-history traits, including immunity [14, 16]. Trade-offs among traits may occur even under the controlled, nutrient-rich environments of modern laying production systems. This is because the “availability of resources” does not refer exclusively to the resources that the animal has access to, but also to the resources that the animal is able to ingest, assimilate, use, and share among functions. The genetic improvement of feed efficiency in modern chickens has contributed to dramatic reductions in the amount of feed required to reach a given body weight or egg production level. In this context of enhanced feed efficiency, feed resources may be limited and trade-offs may be expected when a negative dependency between resource acquisition and resource allocation exists [17, 18].

Most research on trade-offs has been conducted on wild animal species [4], with less interest directed toward domestic species. Experimental selection is commonly used to study evolutionary trade-offs in many species, but, although the methods of artificial selection have been widely applied to livestock, this approach has seldom been used to explicitly study trade-offs in this context [19]. One of the primary goals of selection experiments is to maximize the divergence between control and selected lines or between two divergently selected lines. In poultry, experimental lines that have been selected for either growth or immune traits have proven to be valuable animal models for assessing trade-offs. In 2011, van der Most et al. [13] conducted a meta-analysis that addressed the relationship between growth and immunity; specifically, they examined data from 14 studies on two different chicken lines and one turkey line selected for high body mass. The authors concluded that intensive selection for growth had a large negative effect on resistance to infection and/or immune functions. In contrast, no significant detrimental effect was detected for production traits when selection targeted immune traits such as increased antibody production, improved cell-mediated immune responses, or high phagocytic activity. This prompted the authors to postulate that it should be feasible to select for immune/robustness traits in commercial livestock species without compromising productivity. However, other studies have obtained conflicting results, reporting that increased immune responsiveness (i.e. high antibody production) had a negative long-term effect on growth and egg production [20, 21]. Overall, the relationship between production and immunity in livestock is still unclear and merits further investigation.

The development and maintenance of a fully competent immune system, as well as the mobilization of immune responses to external stimuli, are processes that have a metabolic cost [22–24]. Hence, the immune deficiencies observed in animals selected for high production and efficiency could also suggest the existence of trade-offs involving other energy-demanding processes such as growth, reproduction and thermoregulation [23, 25, 26]. Understanding the nature of trade-offs between production, immune responses, and resistance/susceptibility to disease is of paramount importance in improving animal robustness and welfare and reducing the (ab)use of antimicrobial drugs in animal production.

In this study, we used unique experimental lines of layer chickens that have been developed by the French National Research Institute for Agriculture, Food, and the Environment (INRAE) to investigate: (i) the impact of selection for improved feed efficiency on animals’ immunocompetence, and, (ii) the other way around, the impact of selection for improved humoral vaccine response on animal growth parameters and other aspects of the immune system. In the first experiment, we used two chicken lines (R+ and R−) that have been divergently selected for more than 40 years based on residual feed intake (RFI) at adult age [27, 28]. RFI is a statistically-built index of feed efficiency that represents the deviation of the observed feed intake (FI) of an animal from the feed intake predicted from maintenance and production requirements. Throughout this paper, “R+” refers to the line selected for high values of RFI (low feed efficiency) and “R−” is the line selected for a low RFI (high feed efficiency). RFI was first proposed by Byerly in 1941 [29] as an approach to limit feed costs in laying hens, and was later applied to other species [30]. Although the definition of RFI is consistent among species [31–33], it cannot be considered as a single, invariable trait because both the traits used to estimate the predicted FI and the methods of estimation may differ [34].

In the second experiment, we compared the ND3 line, which has been selected for more than 25 years for increased antibody titers to an inactivated Newcastle disease virus (NDV) vaccine (response measured 3 weeks after vaccination that is performed at 3.5 weeks of age) [35], with its control (CTR) line. Using the layer lines divergently selected for feed efficiency, we addressed the question of whether improved feed efficiency might be detrimental to immunity. Reciprocally, by using the line selected for higher NDV antibody production, we investigated whether an improved humoral immune response may compromise growth, production, and/or other immune system components, such as innate and/or adaptive cellular immune responses.

## Methods

### Animals and sampling procedures

**Fig. 1:**
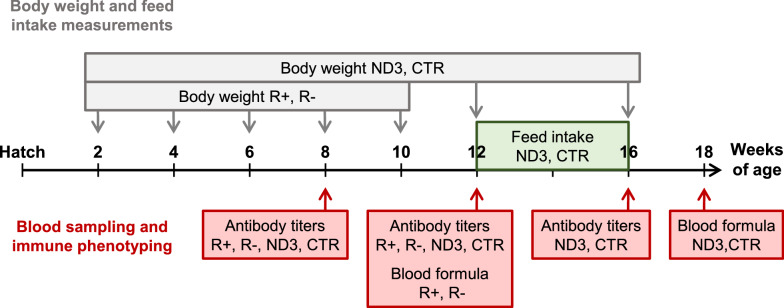
Schematic representation of the experimental design. Gray arrows indicate the ages at which body weight measurements were performed. Red arrows indicate when blood samples were collected for antibody titer quantification and/or leucocyte counts. The type of measurements and the chicken line concerned are indicated in the boxes

R+, R−, ND3, and CTR birds were produced and reared at the INRAE UE1295 PEAT Poultry Experimental Facility (2018, https://doi.org/10.15454/1.5572326250887292E12). All animal experiments were conducted in compliance with European Union Guidelines for animal care; our protocols were approved by the local ethics committee for animal experimentation (Val de Loire) and by the French Ministry of higher education, research and innovation (authorization # 4148-2016). All animals were vaccinated following the standard vaccination protocol applied at the INRAE PEAT facility (Table 1). A comprehensive summary of measurements and blood samplings is given in Fig. 1.

**Table 1:** Vaccination program applied to the R+, R−, ND3, and CTR chicken lines

Vaccination	Age (days)	Commercial name	Delivery mode
Marek’s disease (MDV)	1	MD-VAC^®^ LYO	Subcutaneous
Infectious bronchitis (IBV)	1, 14, 36, 76	Nobilis IB 4/91	Nasal spray
Gumboro disease (IBDV)	21, 28	Avipro Gumboro VAC	Drinking water
Newcastle disease (NDV)	36, 84^a^	Nobilis BI MAS 5-Clone 30	Nasal spray
Metapneumovirus (MPV)	36	Nobilis Rhino CV	Nasal spray
Infectious anemia (CIAV)	63	Avipro Thymovac	Drinking water
Avian encephalomyelitis (AEV)	87^a^	MYELOVAX^®^	Drinking water

^a^These vaccinations were given exclusively to the ND3 and CTR lines

#### R+ and R− lines

The R+ and R− chicken lines are the product of a divergent selection experiment that started in 1976, from a Rhode Island Red population of six sires and 50 dams as described in Bordas and Merat [28]. In this experiment, adult birds were divergently selected based on their RFI value, as measured over a 4-week period (generally from 30–33 to 34–37 weeks of age). Chickens with a low RFI value (R−) are more efficient producers because they require less feed to reach a given body weight (BW) and level of egg production than chickens with a high RFI value (R+). After 40 generations of selection, the divergence in RFI values between the R+ and R− lines is equivalent to five phenotypic standard deviations (see Additional file 1: Figure S1) and the feed intake of the R+ line is twice that of the R− line [36].

This study used 34 R− chickens (19 males and 15 females), produced from four sires and 20 dams (5 dams per sire), and 37 R+ chickens (24 males and 13 females), produced from three sires and 18 dams (6 dams per sire). These animals belonged to generation 40 of divergent selection and were hatched and reared together in floor pens under standard conditions until 12 weeks of age, with ad libitum access to water and feed. From hatching to 9 weeks of age, birds were fed a starter diet containing 19% crude protein (CP), 3.4% crude fat (CF), 0.9% calcium (Ca), 0.6% total phosphorus (P), 0.93% lysine (Lys), and 2800 kcal/kg of metabolizable energy (ME). From 10 to 18 weeks of age, birds were fed a grower diet composed of 15.4% CP, 3.4% CF, 1.1% Ca, 0.7% P, 0.7% Lys, and 2750 kcal/kg of ME.

In vertebrates, the major histocompatibility complex (MHC) region plays a pivotal role in the response to infectious diseases, as it contains highly polymorphic genes encoding molecules that present antigens to T cells. MHC is essential in defense against pathogens and central in defining the specificity of the adaptive immune system [37, 38]. To minimize the potential effects of MHC variability on immune responsiveness, all R+ and R− chickens were selected to have the same MHC genotype (homozygous for an allele similar to the reference MHC allele B10); this was verified by genotyping the LEI0258 marker in the parents (all homozygous for the same allele) following the protocol in [39].

All birds were weighed at 2, 4, 6, 8, and 10 weeks of age. Blood was sampled from the wing vein of all birds at 8 and 12 weeks of age to perform leukocyte counts and antibody quantification (Fig. 1). More specifically, at 8 weeks of age, 29 R− (16 males and 13 females) and 21 R+ (10 males and 11 females) chickens were sampled. Between 8 and 12 weeks of age, two male R− birds and one female R+ bird died, leaving 27 R− (14 males and 13 females) and 20 R+ (10 males and 10 females) birds for sampling at 12 weeks.

#### ND3 and CTR lines

The ND3 line is the product of a selection experiment that started in 1994 on a population of commercial White Leghorn chickens and selected for high antibody response to the NDV vaccine 3 weeks after vaccination (performed at 3.5 weeks of age) [35]. The CTR line originated from the same White Leghorn founder population and has been maintained by random mating since then. After 20 generations of selection, the ND3 line has an average NDV antibody titer, expressed on a log scale with a base of 2, twice as high as the unselected CTR line, with a value of 5.75 for ND3 and 2.50 for CTR.

This experiment used 39 female ND3 chickens, produced from 11 sires and 27 dams (2 to 3 dams per sire), and 38 female CTR chickens produced from 12 sires and 22 dams (1 to 3 dams per sire). Because of the presence of line-specific MHC genotypes (Table 2), it was not possible to select ND3 and CTR chickens with the same MHC genotype for this experiment. The birds were reared together in floor pens under standard conditions from hatching to 11 weeks of age, and were then transferred to individual cages equipped with individual feeders to measure individual feed intake from 12 to 16 weeks of age. The birds had ad libitum access to water and feed throughout the experiment. The nutritional composition of the feed was the same as described for the R+ and R− birds.

**Table 2:** MHC genotypes identified in the ND3 and CTR lines

Lines	MHC genotype frequencies (%)							
	B34-B124	B21-B34	B21-B124	B34-B34	B124-B124	B15-B124	B15-B21	B15-B15
ND3	53.3	3.3	3.3	23.3	16.7	0.0	0.0	0.0
CTR	0.0	0.0	3.3	0.0	13.3	53.3	6.7	23.3

Body weight was measured weekly for all animals from 2 to 10 weeks of age and at the start (12 weeks) and the end (16 weeks) of the individual feed intake control period (Fig. 1). We chose not to include these two last time points in the estimation of the growth curve because the animal rearing conditions changed as the birds were moved to individual cages at 11 weeks of age, which represented a source of stress.

Blood samples were collected from 30 ND3 and 30 CTR chickens at 8, 12, and 16 weeks of age for antibody titer assessment and at 18 weeks of age for blood leucocyte counts (Fig. 1).

### Assessment of vaccine-specific antibodies

Antibody titers were measured in serum samples by the LABOCEA laboratory (Ploufragan, France). Antibodies against infectious bursal disease virus (IBDV) and infectious bronchitis virus (IBV) were quantified using direct ELISA tests from IDEXX (Laboratory, Inc., USA) and Biochek (Reeuwijk, The Netherlands), respectively. For these two tests, antibody titers were expressed as the antilog of the log_10_ titer, obtained by calculating the sample to positive ratio (S/P = mean of test sample − mean of negative control/mean positive control) and using the equation log_10_ titer = 1.09 (log_10_ S/P) + 3.36 for IBV, and the equation log_10_ titer = 1.1 (log_10_ S/P) + 3.361 for IBDV, according to the manufacturers’ instructions. Antibodies against chicken infectious anemia virus (CIAV) were quantified using a competitive ELISA test (IDEXX) and expressed as (1 − antibody titer) × 100. Antibodies against avian metapneumovirus (B strain) were quantified using an in-house direct ELISA test developed by the LABOCEA laboratory and expressed as S/P. Antibodies against Newcastle disease virus (NDV) were quantified by testing serial two-fold dilutions in a standard hemagglutination inhibition assay and were expressed as log_2_ values_._

### Leucocyte quantification in whole blood

The absolute number of cells of different leucocyte sub-populations in peripheral blood was assessed at 12 weeks of age for the R+/R− birds and at 18 weeks of age for the ND3/CTR birds using a high-precision flow cytometry-based protocol [40]. A list of the cell types tested and their respective immune functions is in Table 3. Blood was collected from 20 R+ (9 males and 11 females), 27 R− (14 males and 13 females), 30 ND3, and 30 CTR (all females) individuals in EDTA-coated tubes; 200 µL of each sample were transferred to a 0.5-mL reaction tube and stabilized with 40 µL of TransFix^®^ reagent (Invitrogen). Samples were analyzed by flow cytometry using the no-lyse/no-wash/single-step/one-tube method. Briefly, diluted whole blood was incubated for 45 min at room temperature in the dark with fluorochrome-labeled monoclonal antibodies and then analyzed with a BD FACS Canto II for quantification, using BD TruCount tubes™. Combined staining to identify thrombocytes, monocytes, heterophils, B cells, and T cells was performed with anti-chCD4-FITC (clone CT4), anti-chCD8a-FITC (clone CT8), anti-chTCRγδ-FITC (clone TCR1), Kul01-RPE, anti-chBu1-PerCP-Cy5.5 (clone AV20) (all from SouthernBiotech, Birmingham, USA), K1-RPE [41], and anti-chCD45-APC (clone UM16-6, Bio-Rad Laboratories, Hercules, USA). A second staining was performed to discriminate among subpopulations of T cells, using anti-chCD8-FITC, anti-chTCRγδ-PE, anti-chCD4-PerCP-Cy5.5, and anti-chCD45-APC. Conjugation of mAb K1 to RPE, mAbs CT4 and AV20 to PerCP-Cy5.5, and mAb AV20 to APC was performed using the respective Lynx Rapid Conjugation Kits^®^ (Bio-Rad Laboratories, Hercules, USA) according to the manufacturer’s instructions. Data were analyzed with FlowJo (Tree Star Inc., OR, USA) software. A gating strategy was used to eliminate possible contamination with other cytotoxic lymphocytes, such as NK cells from the CD8^+^ lymphocyte gate. We considered the number of NK cells in the CD8^+^ lymphocyte population to be negligible, and, unlike what is typically observed in the spleen and intestinal mucosa, NK cells do not appear to display significant CD8^+^ molecules in blood [14, 42, 43]. Some aberrantly low values were obtained for blood CD8α^+^ cell numbers in 10 ND3 and 5 CTR samples; this was likely the result of an immuno-labeling issue linked to a polymorphism in CD8α, the existence of which in chickens was suggested by Luhtala et al. [44] but has never been tested in these chicken lines. As we used only one commercial chicken CD8α antibody in this experiment, we chose to remove these samples from the analysis of this cell type, but included them in the analyses of all other cell types.

**Table 3:** Identification of immune cell populations in chicken blood and putative functions in anti-viral immunity

Immune system	Blood immune cell populations^a^	Cell surface markers (labeled with monoclonal antibodies)^b^	Functions^c^
Innate	Myeloid cells		Immediate non-specific response
	Heterophils	CD45dim population, no specific marker	Inflammation, phagocytosis, anti-microbial activity
	Monocytes	CD45bright population KUL01+	Inflammation, phagocytosis, anti-microbial activity; antigen presentation
Adaptive	Lymphocytes	CD45bright populations	Delayed response, virus-specific memory triggered after vaccination
	T cells^d^	Including all CD4^+^, CD8α^+^, TCRγδ^+^	
	TCRγδ^+^	CD8α^+^ or CD8α^−^	Interface between innate and adaptive immunity
	TCRγδ^−^		Delayed response, virus-specific memory triggered after vaccination
	Helper	CD4^+^	Help for anti-viral specific antibody and cytotoxic responses
	Cytotoxic	CD8α^+^	Anti-viral specific cytotoxic responses and IFN-γ production
	B cells	BU1^+^	Specific antibody (neutralizing) production

^a^FACS analysis was performed according to Seliger et al. [40]. NK cells and myeloid dendritic cells were not tested because they are not well characterized in chicken blood and are assumed to be rare (less than 1% of leucocytes)

^b^References for monoclonal antibodies are in Seliger et al. [40]

^c^Functions of immune cell populations are indicative. More details are available in the book *Avian Immunology* [96]

^d^Expressions of the T cell receptor (TCR) αβ and γδ are mutually exclusive. TCRαβ is involved in antigen peptide recognition after presentation via MHC I/II molecules. TCRγδ is involved in recognition of a variety of antigens (including peptides, lipids, glycol-lipids, and phospho-antigens). The FACS strategy to separate helper and cytotoxic T cells in chicken blood was based on the expression or the absence of expression of TCRγδ [40]. The CD4^+^ helper T cell subset expresses only TCRαβ (not tested). CD8α^+^ T cells express TCRγδ or TCRαβ (not tested), the latter of which includes anti-viral cytotoxic T cells. TCRγδ^+^ T cells include CD8α^+^ and CD8α^−^ T cell subsets that may display cytotoxic activity against tumor cells and/or IFN-γ production

### Statistical analysis

Prior to modeling, all quantitative variables were tested for normality with the Shapiro–Wilk test [45] using the *shapiro.test* function in R [46]. Non-normal variables (i.e., IBV antibody titers) were subsequently log10 transformed; however, to facilitate interpretation, all tables present least square means (± SE) on the scale of untransformed variables.

#### Analysis of immunity traits

For each immunity trait, a linear mixed model including fixed and random effects was fitted as described below. To account for the potential relationship between growth and immunity, we included the average weekly gain (AWG) in body weight as a covariate in the model for the R+ and R− lines from 2 to 10 weeks of age, as well as in the model for the ND3 and CTR lines from 2 to 16 weeks of age. For the R+ and R− cell-count data, the line, sex, and their interaction were included as fixed effects. For the R+ and R− antibody titer data, the interaction was not significant and was thus not included in the final model. For the ND3 and CTR cell and antibody data, the mixed model included only the line as a fixed effect. In all cases, a random sire effect was included to account for family structure. These linear mixed models were fit using the *lme* function from the *nlme* R package [47], and Wald Chi-square tests for fixed effects were estimated using the *Anova* function from the *car* package [48]. Least square means and adjusted p-values were calculated using the *emmeans* R package [49], and pairwise differences between lines were evaluated using Tukey’s honest significant difference test (using a significance threshold for adjusted p-values of α = 5%).

#### Analysis of growth patterns

To compare growth patterns between lines, a linear mixed model that included line, age, and their interaction as fixed effects, and the animal as a random effect was fitted. Sex was added as a fixed effect for the analysis of the R+ and R− lines. To account for within-individual correlation, a first-order autoregressive error structure was fitted to the model using the *corAR1* function in the *nlme* R package [47].

#### Estimation of residual feed intake

In the ND3 and CTR lines, RFI was calculated as the difference between the observed FI and the predicted FI (PFI) for the recorded period: (RFI = FI − PFI). PFI was estimated by a multiple regression equation calculated for all animals using two independent variables: average BW and BW gain between 12 and 16 weeks of age [27, 31]. The estimated feed conversion between 12 and 16 weeks represents the ratio between the total feed intake and the BW gain over the recorded period.

#### Multivariate analysis of cell-type and antibody differences between R+ and R− lines

To identify the cell types and antibodies with the largest contributions to the differences between the R+ and R− lines, we used a between-group principal component analysis (BGA) [50] and a permutation test to check the significance of separation among groups. The BGA analysis consists of a supervised extension of the PCA, and its purpose is to order the groups by maximizing between-group variance. This method visually assesses the differentiation between groups and identifies the variables that contribute the most to this differentiation. This analysis was performed with the *bca* function of the *ade4* package [51].

## Results

### Comparison between R+ and R− chicken lines

#### Growth rate

**Fig. 2:**
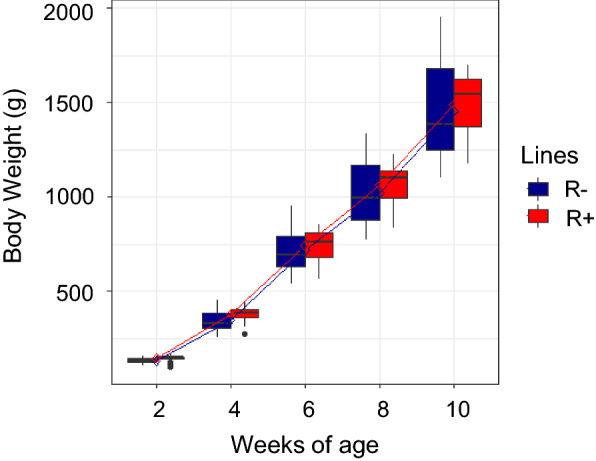
Growth curve of R+ and R− chickens measured from 2 to 10 weeks of age. No significant difference was observed between lines

Line had no significant effect on weekly body weight gain (160 g for R+ and 162 g for R−; p = 0.8). Similarly, no significant line × age interaction was observed (p = 0.5), indicating a linear trend for weight gain (Fig. 2).

#### Vaccine-specific antibody response

**Fig. 3:**
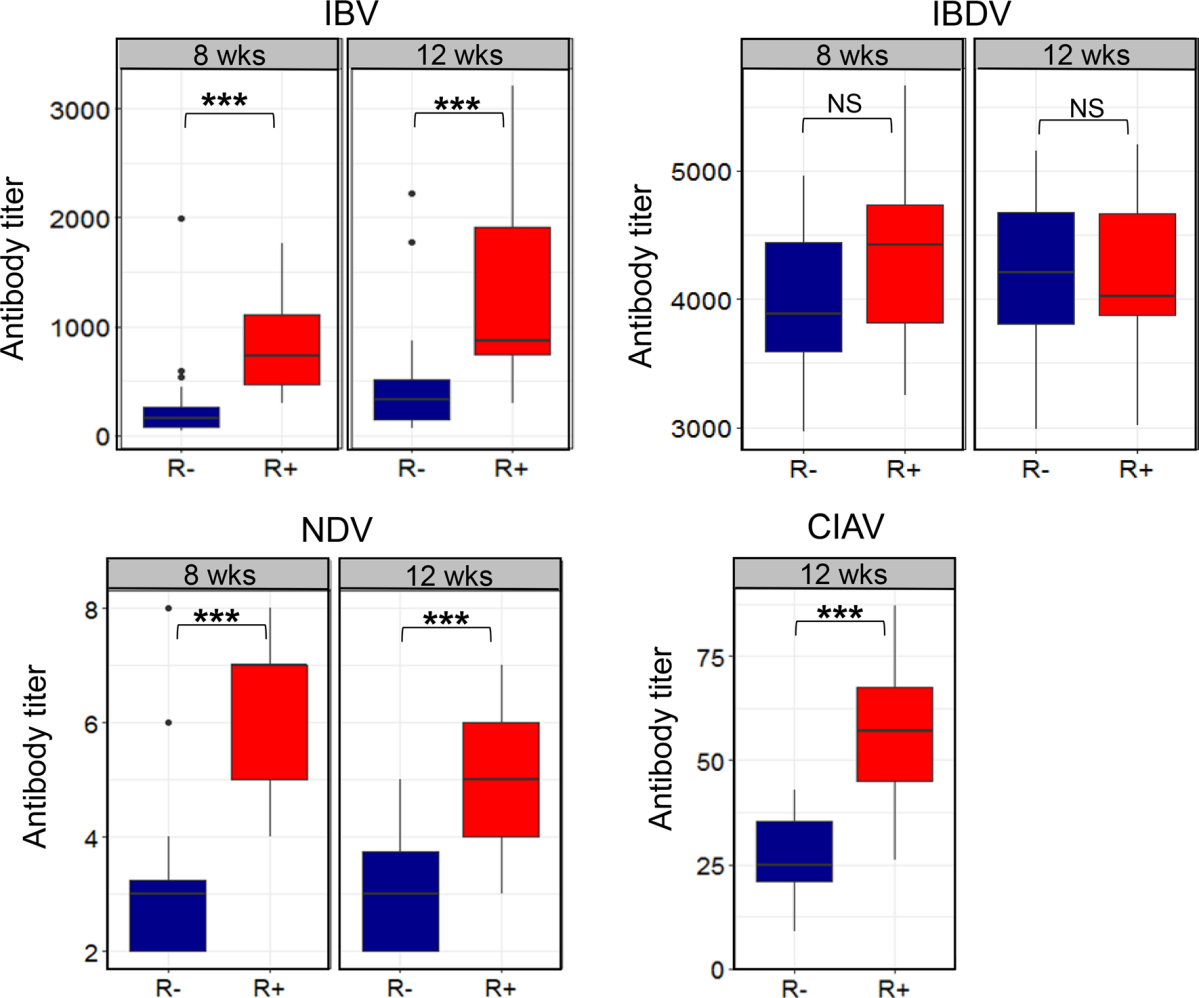
Humoral antibody titers of R+ and R− chickens, measured by ELISA or IHA (NDV) after vaccination, at 8 and 12 weeks of age. Asterisks indicate significant differences between lines (*p < 0.05; ***p < 0.001), NS = not significant

No significant covariation was observed between AWG and antibody titers (see Additional file 2: Table S1). There was a significant effect of line (p < 0.01) on all vaccine-specific antibody titers except IBDV (Fig. 3) and (see Additional file 2: Table S1). Specifically, the R+ chickens had a stronger antibody response at 8 and 12 weeks of age to all viral vaccines. Indeed, at both time points, IBV and NDV antibody titers were on average three times higher in the R+ line than in the R− line. There was no effect of sex on any of the vaccine antibody responses tested (see Additional file 2: Table S1), indicating that the humoral immune response did not differ between males and females.

#### Blood leucocyte counts

We detected substantial variation between the R+ and R− lines for several blood cell types (Table 4). The R+ line presented larger numbers of circulating heterophils and macrophages (p < 0.001), while R− chickens had larger numbers of circulating helper T cells (phenotypically defined as CD45^+^ BU1− lymphocytes) (p < 0.05) and marginally larger numbers of cytotoxic T cells (p = 0.09). B and T cells demonstrated sex-specific patterns (p < 0.05), with larger numbers in females than in males (from + 22% to + 50%, depending on the cell type); this difference was more pronounced in R− than R+ chickens (Table 4). A significant interaction between line and sex (p < 0.01) was observed for monocytes: in the R+ line, higher cell counts were observed in males (on average 22% more than in females), while in the R− line higher cell counts were observed in females (on average 32% more than in males). AWG was found to have no significant effect on leucocyte counts (p > 0.1 for all models).

**Table 4:** Whole blood leucocyte counts (× 10^3^ cells) in vaccinated R+ and R− chicken lines measured at 12 weeks of age

	Heterophils	Monocytes	T cells	B cells	CD4^+^ helper T cells	CD8α^+^ T cells	CD8α^+^γδ^−^ cytotoxic T cells	CD8α^+^γδ^+^ T cells	γδ^+^ T cells
Line/sex^a^									
R+ m	9.1 ± 0.8^a^	3.3 ± 0.3^a^	23.8 ± 4.0	0.9 ± 0.4	8.2 ± 0.1	4.6 ± 0.5	3.5 ± 0.4	1.3 ± 0.1	6.0 ± 0.5
R+ f	7.5 ± 0.6^a^	2.3 ± 0.2^ac^	26.9 ± 3.7	1.1 ± 0.3	9.6 ± 0.8	4.7 ± 0.4	3.8 ± 0.3	1.1 ± 0.1	6.1 ± 0.4
R− m	3.2 ± 0.6^b^	1.3 ± 0.2^b^	22.0 ± 3.2	0.6 ± 0.3	10.5 ± 0.7	5.6 ± 0.4	4.6 ± 0.3	1.1 ± 0.1	5.9 ± 0.4
R− f	3.4 ± 0.6^b^	1.6 ± 0.2^bc^	28.6 ± 3.3	1.2 ± 0.3	10.7 ± 0.8	5.0 ± 0.4	4.0 ± 0.3	1.0 ± 0.1	6.1 ± 0.4
p-value^b^									
Line	***	***	0.9	0.9	*	0.1	0.09	0.2	0.9
Sex	0.3	0.3	*	*	0.4	0.4	0.6	0.2	0.8
Line × sex	0.1	**	0.3	0.4	0.3	0.3	0.1	0.8	0.9

Within a column, least square means with different superscripts differ significantly (p < 0.05)

m = male; f = female

^a^Values are least square means (± SE) of cell counts per µL of whole blood

^b^Wald Chi-square test was significant at p < 0.05*; p < 0.01**; p < 0.001***

#### Between-group analysis of cell counts and antibody titers

**Fig. 4:**
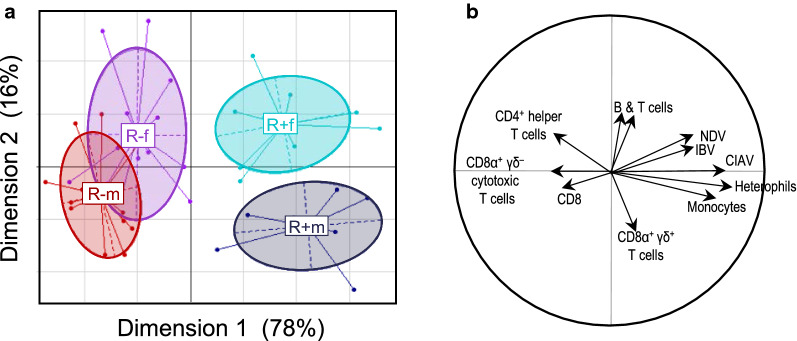
**a** Projection of first and second axis of BGA based on white cell count and antibody titer data. Individuals are grouped and color-coded according to line (R+ and R−) and sex (m = males and f = females): R− m = red, R+ m = blue, R− f = purple, and R+ f = turquoise. **b** Representation of the partial contributions of cell types and antibodies to differentiation along the first two axes

Figure 4a, b depict, respectively, the factorial plot of the first two axes of the between-group analysis and the corresponding correlation circle, which shows the correlations of different variables with these two axes. Significant separation was observed between both lines and sexes (p = 0.01). The first axis explained most of the between-class variability (78%) and separated the groups based on genotype (R− on the left side and R+ on the right side of the graph). The second axis explained 16% of the between-class variability and separated the males from the females. The cells and antibodies that contributed the most to differentiation between lines were monocytes, heterophils, CIAV, IBV, and NDV, which were higher in the R+ group, and CD4^+^ helper T cells and CD8α^+^γδ^−^ cytotoxic T cells, which were higher in the R− group. The cell types that contributed the most to the differences observed along the second axis (separating males from females) were T and B cells and CD8α^+^γδ^+^ T cells. No contribution from the antibodies was observed for the second axis.

### Comparison between ND3 and CTR chicken lines

#### Growth rate and feed intake

A lower AWG in body weight was observed for the ND3 line compared to the CTR line (least square means (± SE) of AWG (g): 105 ± 1.7 versus 114 ± 1.7, p < 0.001, with a difference of − 9 ± 2.6).

**Fig. 5:**
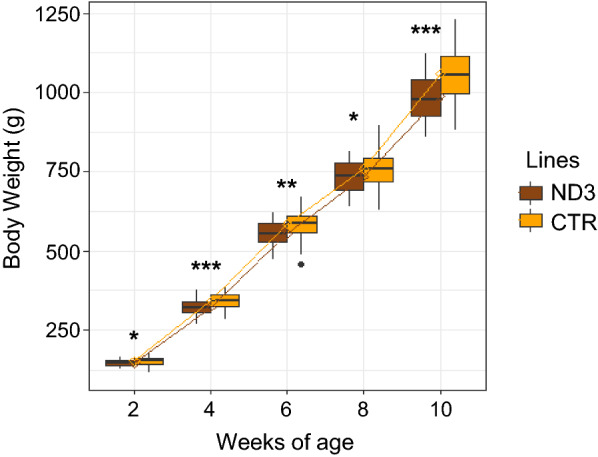
Growth curve of ND3 and CTR chickens measured from 2 to 10 weeks of age. Asterisks indicate significant differences between lines at a given age (*p < 0.05; **p < 0.01; ***p < 0.001)

**Fig. 6:**
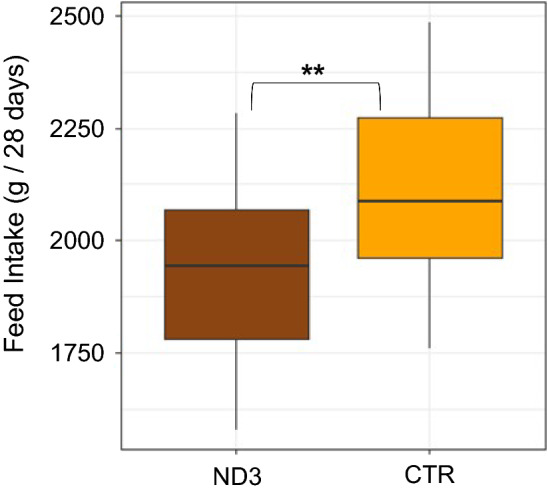
Feed intake of ND3 and CTR chickens measured over 28 days, from 12 to 16 weeks of age. Asterisks indicate significant differences between lines (***p < 0.001)

Line had a significant effect on body weight at all ages (p < 0.05 for all ages), with ND3 chickens having significantly smaller body weights (on average between 3 to 7% smaller) compared to CTR chickens. A significant line × age interaction was observed (p < 0.001), indicating a difference in growth rate between the two lines (Fig. 5). Feed intake differed significantly between lines (p = 0.002), with the ND3 birds eating less than the CTR birds (Fig. 6). No differences between lines were observed for residual feed intake (p = 0.3) and feed conversion rate (p = 0.9).

#### Vaccine-specific antibody response

At the three ages tested (8, 12, and 16 weeks of age), the ND3 line had significantly higher antibody titers against all viral vaccines administered, with the exception of MPV antibodies at 8 weeks of age and CIAV antibodies at all ages tested (Table 5). As expected, the largest difference was observed for the NDV vaccine (selection criterion for the ND3 line), with the ND3 chickens presenting antibody titers that were three to four times higher than those of the CTR birds. The ND3 line also produced about twice as many specific antibodies than the CTR line in response to IBV and IBDV vaccination at the three time points, while for MPV the difference ranged from + 10 to 20% at 12 and 16 weeks of age.

**Table 5:** Humoral antibody response after vaccination in ND3 and CTR chicken lines

	IBV			IBDV			NDV			CIAV		MPV		
Weeks	8	12	16	8	12	16	8	12	16	12	16	8	12	16
Lines^a^														
ND3	1592 ± 161	1023 ± 97	774 ± 99	893 ± 43	1128 ± 123	1136 ± 72	6.6 ± 0.3	6.3 ± 0.2	7.8 ± 0.3	55 ± 7.7	92 ± 2.8	0.1 ± 0.04	1.1 ± 0.02	1.1 ± 0.04
CTR	651 ± 162	442 ± 99	485 ± 99	562 ± 42	673 ± 120	872 ± 71	4.3 ± 0.3	4.4 ± 0.2	6.1 ± 0.3	56 ± 7.6	86 ± 2.7	0.09 ± 0.04	0.9 ± 0.02	1.0 ± 0.04
p-value^b^														
Line	***	***	*	**	**	*	***	***	***	0.9	0.2	0.4	***	*

Vaccine-specific antibody titers in sera were measured by IHA test (NDV) or ELISA tests, either direct (IBV, IBDV, MPV) or competitive (CIAV), at 8, 12, and 16 weeks of age

^a^Values are least square means of antibody titers (± SE)

^b^Wald Chi-square test was significant at p < 0.05*; p < 0.01**; p < 0.001***

#### Blood leucocyte counts

**Fig. 7:**
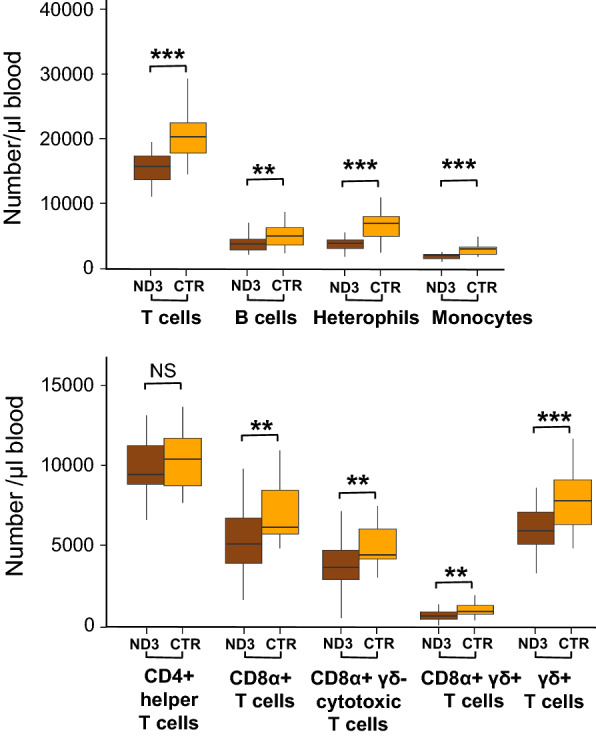
Absolute white blood cell counts in ND3 and CTR chickens, expressed in number of cells per microliter of blood. Asterisks indicate significant differences between lines (**p < 0.01; ***p < 0.001), NS = not significant

Line had a significant effect (p < 0.01) on the cell counts of most types of white blood cell types (Fig. 7) and (see Additional file 3: Table S2). Compared to the ND3 line, the CTR line had approximately 50% more heterophils and monocytes and 30% more B and T cells overall, with the same pattern reflected in CD8α^+^, CD8α^+^γδ^−^, and CD8α^+^γδ^+^ T cells. The only cell subpopulation that did not differ between lines was the CD4^+^ helper T cells (p = 0.4).

## Discussion

Artificial selection is a useful way to experimentally investigate a given trait’s potential for improvement and the implications of such changes for other traits. Our results indicated that two experimental lines of chickens that have been divergently selected for high and low RFI, respectively, differed with regard to both humoral and cellular immune traits. Conversely, the ND3 line, which has been selected for high antibody production for one specific viral antigen, showed improved immune reactivity for a wide range of viral vaccines, but a smaller number of circulating leucocytes and reduced feed intake and growth compared to the control line.

The numerous differences observed between lines could be indirect correlated effects of selection; however, considering the small population size and the relatively high level of inbreeding in our experimental populations, these effects could also be due to genetic drift. Genetic drift causes stochastic fluctuations in allele frequencies across the genome, in contrast with selection-driven changes in allele frequencies, which are directional and region-specific. Selection and genetic drift do not act in isolation, and trait variation is most likely a result of both mechanisms. For example, the MHC variation in the ND3 and CTR lines may represent this double contribution. In our study, we observed a loss of MHC variability in both lines compared to the initial, pre-selection population [35]. A loss of MHC haplotypes in the CTR line may seem surprising considering that this line is not under selection. The most plausible explanation is that this is the result of genetic drift over the years, accentuated by the small effective population size of the line [52]. In the ND3 line, the loss of some MHC haplotypes and an increase in frequency of others was rapidly detected during the first few generations of the experiment [35]; this was interpreted as the effect of selection for specific alleles that have a positive effect on ND3 antibody production, estimated to be 2.3% of the total phenotypic variation [52].

Considering the intensive selection pressure to which the R+, R−, and ND3 lines were exposed over many generations, we think that it is likely that the differences observed between lines are in large part the correlated effects of selection rather than the extensive effect of random drift. This consideration is supported by the fact that our results, particularly those from the ND3 and CTR lines, are in agreement with those obtained in previous studies in chickens and other species [53–55], in which negative phenotypic correlations between growth and immune responses were observed. Such widespread agreement would be highly improbable in a scenario that relied exclusively on drift. Our hypothesis is that the differences observed between lines could be the result of trade-offs between functions, potentially involving energetic and/or non-energetic-based mechanisms.

Among the many biological functions of an organism, the immune system makes a major contribution to individual fitness but is heavily dependent on metabolic resources for proper functioning. Thus, it is in competition with other nutrient- and energy-demanding processes in the organism’s resource-allocation strategy [25]. The metabolic cost of an immune response results from the amount of energy invested in the activation process and in the increase and turnover of immune cell/protein pools [56, 57]. Mounting an immune response that involves both the innate and adaptive systems is considered energetically costly due to both the direct and indirect metabolic requirements of each one [22–24]. In humans, even relatively mild immune activity is accompanied by an 8 to 14% increase in metabolic rate [58] and requires the reallocation of energy from storage, growth, and development towards the immune system [59]. Because the immune system is a complex network of many cell types and accessory proteins, direct quantification of its cost remains difficult [25]. In chickens, Klasing [60] estimated the nutritional resources needed for immune response compared with those dedicated to normal growth by examining the contribution of leukocytes and accessory proteins to body mass. He showed that the normal daily production of leucocytes and immunoglobulins in adult chickens contributes slightly less than 1% of body weight. Through experiments that have evaluated the effects of antibiotics on growth or compared chicken growth in a sterile environment compared to a conventional, pathogen-free environment, the maintenance costs of immunity have been indirectly estimated to be in the range of 5% of daily nutrient needs [61, 62]. These results confirm that the immune system at rest has a measurable cost, but is not a significant consumer of nutritional resources [61]. However, the immune system is not the only system that increases its nutrient requirements as a result of pathogen exposure. Many infections, including those related to vaccination, trigger an acute phase response, which is characterized by increased protein synthesis and can account for about 9% of the body’s total nutrient use [63].

It is beyond the scope of this study to investigate the underlying mechanisms of the trade-off between functions. Instead, this work reveals the existence of phenotypic relationships between feed efficiency, immune function, and growth traits, and puts forward hypotheses about the potential energy-allocation strategies adopted by the experimental lines investigated here.

### Impact of long-term selection to improve feed efficiency on animal growth and the humoral response to vaccination

Previous work has identified other important physiological traits that are correlated with selection for high or low feed efficiency in adult chickens [27], including a larger body fat content in R− birds [64] and a higher body temperature and diet-induced thermogenesis in the R+ line [65, 66]. These differences support the hypothesis that selection for high or low feed efficiency has effectively altered energy metabolism and nutrient utilization between these two lines [67, 68]. In our study, we did not determine RFI in young birds, but previous results from generations 15 and 18 of the R+/R− lines revealed that differences between the lines are also evident in juveniles [69]. More specifically, in females, significant differences in RFI were detected between lines as early as 8 weeks of age and in FI as early as 6 weeks of age, suggesting that metabolic changes between lines appear well before the age targeted by selection. This early differentiation between lines is also supported by an ongoing whole-genome transcriptomic study comparing liver gene transcription profiles between the R+ and R− lines at 12 and 26 weeks of age. Preliminary results indicate that there are large differences in expression between the lines at both ages, with 1000 differentially expressed genes at 12 weeks of age (Zerjal et al., personal communication). With respect to body weight, Bordas and Minvielle [69] observed clear differences between lines: as juveniles, male R− birds were heavier than their R+ counterparts, while R− females were heavier than R+ females from 4 to 30 weeks of age. This prompted the authors to argue that R− chickens were more efficient growers. However, in the current study we observed no difference in BW between the two lines from 2 to 10 weeks of age. These contrasting observations might be explained by the fact that the R+/R− birds used here (generation 40) are the product of 20 additional generations of selection. In those intervening generations, the progress obtained in R− birds can be summed up as an additional reduction of 80% in RFI and 8% in FI in females, and of 62% and 21% in males, respectively [36] and (see Additional file 1: Figure S1). It is conceivable that, at the present stage, the level of efficiency of the R− birds has reached such a critical point that, in order to ensure the proper functioning of maintenance processes, the organism must slow down other functions, such as body growth.

In our study, after repeated vaccinations against common viral diseases, there were clear differences between the R+ and R− lines with respect to vaccine-specific antibodies and cellular responses between 8 and 12 weeks of age. Compared to R− birds, R+ birds displayed a higher capacity to mount a specific antibody response to live attenuated viral vaccines, which suggests that R+ chicks are better responders with respect to adaptive humoral immunity.

Interestingly, these results conflict with previous findings of Van Eerden et al. [70], who reported no significant differences between feed-efficient and -inefficient birds in antibody responses against various antigens (keyhole limpet hemocyanin, *M. butyricum*, and *S. enteritidis* LPS). This discrepancy may be due to two major factors. First, the immunization protocol of the previous study was based on the use of inert model antigens and may not have been effective enough to reveal differences in avian antibody response capacities. Second, the birds used in that study represented the extremes of the RFI distribution from a conventional population of Lohmann Brown layer chickens that differed only moderately in RFI, which might explain the lack of difference in antibody production.

Our results also differ from those obtained on young pigs that were divergently selected to obtain high- and low-RFI lines [71]. These pig lines differed only moderately in their immune and metabolic responses to an inflammatory challenge, and no disadvantage was observed for the low RFI line. There are very few studies that have investigated the relationship between RFI and immune traits, and general conclusions cannot be drawn. What is certain is that the biology underlying RFI is complex, and it is inappropriate to refer to RFI as a single, invariable trait across species. Selection for low or high RFI will not necessarily affect the same functions among different species, as becomes clear with comparisons of trait correlations. For example, in pigs the low-RFI line is leaner [71], while the opposite is true for R− chickens [64] indicating that selection for improved feed efficiency has clearly affected different functions in the two species.

### Impact of long-term selection for improved feed efficiency on blood leucocyte numbers

Our analysis of white blood cells revealed differences between the R+ and R− lines and/or between sexes. T and B cell counts were unaffected by line, but females had higher cell counts than males. This is in agreement with previous work in commercial broiler lines, in which females had higher blood lymphocyte counts than males [72]. Since, in our study, the male and female birds were reared together, differences in lymphocyte counts cannot be attributed to different environments or pathogen exposure, but rather suggest sex-based differences in the disposition of the immune system. As yet, we do not have an evident explanation for this difference, but sexual dimorphism in immune functions seems to be a relatively common pattern in vertebrates, with females generally displaying a greater investment in immunity than males [73].

Differences between lines were apparent with respect to blood T cell subpopulations. The R− chickens displayed more circulating CD4^+^ helper T cells than R+ chicks did, and marginal differences (p = 0.09) were also observed for CD8α^+^γδ^−^ cytotoxic T cells. This result, combined with the higher antibody response observed in the R+ chickens, suggests the existence of different immune activation profiles in the two lines, with R− chickens being more oriented towards type I (cell-mediated immunity) and R+ chickens being more dependent on type II (antibody-mediated immunity) immune responses after vaccination. These findings may seem surprising considering the metabolic costs that are generally allocated to the cellular and humoral arms of the adaptive immune response: activation of the innate response (cell-mediated immune responses that result in the production of pro-inflammatory cytokines) is considered to be more costly than the adaptive immune response (antibody-producing humoral responses relying on anti-inflammatory cytokines) [56, 63]. Although this is speculation, we could hypothesize that shifting to immune memory through the process of adaptive immunity might be a way to reduce, in the long term, the global cost of immune responsiveness, as has already been proposed in other studies [74, 75].

Strikingly, there was a marked difference in the number of heterophils and monocytes between lines, with R+ chickens showing more circulating heterophils and monocytes than the R− birds. This may indicate that the long-term selection on RFI has had consequences also for the innate immune system. Heterophils and monocytes constitute major phagocytic cellular components in chicken blood [76–78] and play an important role in the innate immune system as mediators of acute inflammatory responses. It is possible that the larger number of circulating monocytes and heterophils observed in R+ chickens predispose these birds to develop inflammation, which may be beneficial or detrimental depending on the environmental/pathogen exposure in question. Given that optimal immune functioning does not necessarily correlate with maximum immune reactivity [79, 80], the elevated number of professional phagocytes in the R+ line clearly merits further study regarding phagocytic capacity and its potential role in the clearance of bacterial infections.

In agreement with previous observations, the selective pressure to improve feed efficiency has also affected immune-related organs, with R+ chickens displaying heavier spleens that contain a larger number of T and B cells (T. Zerjal and P. Quéré, unpublished observations). The spleen is the major lymphoid organ involved in antigen presentation in chickens [81]; the increase in spleen size and in total numbers of T and B cells per spleen may result in higher efficiency in the vaccine antibody response in R+ birds. Although a large part of the excess ingested energy in these chickens is dissipated as metabolic heat [66], resource levels are still high and available for other important functions. This surfeit of available energy in R+ birds might have promoted immune-related organ development and a corresponding increase in immune cell proliferation and immune reactivity, in line with a better antibody response.

### Impact of long-term selection for increased antibody production on blood leucocyte numbers

Compared to control birds, the ND3 line presented a significant reduction in most circulating leucocyte populations and sub-populations, including B cells, which may seem surprising considering that these cells are directly responsible for antibody production. At this stage, we cannot tell whether differences in immune cell counts also exist at the level of immune organs, because we lack data on immune organ weights and the related immune-cell population counts for the ND3 and CTR lines. However, evidence obtained in mammals indicates that white blood cell counts do not necessarily correlate with white blood cell (re-)activity [82]. Previous studies with chickens demonstrated that birds selected for high antibody responses had macrophages with greater phagocytic capacity than those selected for low antibody responses [83, 84]. This was linked to a stronger antigen-presenting ability of macrophages from the high antibody response line, which would also explain its higher capacity for producing antibodies [85].

### Impact of selection for high antibody production on animal growth

Previous studies on chickens have been fairly unanimous in indicating that selection for rapid growth has a negative impact on immune function [14, 86, 87]. In contrast, data on the impact of selection for increased immune responses on animal growth are scarce and rather ambiguous, with large differences among lines [13]. To investigate the impact of improved humoral immune response on chicken growth, we compared the ND3 line to its unselected control line. Our results show that the ND3 line had a stronger antibody response against a large panel of live-virus vaccines but presented a reduced growth rate and reduced (− 9%) feed intake. To further analyze the relationship between immune traits and growth, we included as a covariate in our model the average weekly gain. No covariation was observed between growth and immunity, which indicates that there was no evidence for an ongoing trade-off between them. However, since the ND3 line has undergone many generations of selection for increased antibody production, its reduced body weight compared to the control line might result from previous trade-offs built up with time. When searching for trade-offs through analyses of covariation, it might be more profitable to consider measurements taken at early stages of selection, when direct and correlated responses are expected to be stronger.

The strong immune activation observed in response to vaccination in juvenile ND3 birds may be responsible for a reduction in appetite that eventually affected growth; however, this reduced feed intake was not observed in adult ND3 birds, in spite of a reduced body weight when compared to CTR chickens (data not shown). Other studies have reported the existence of a negative correlation between antibody response and body weight in layer chickens, specifically in lines selected for divergent responses to intravenous or subcutaneous injection of a sheep red blood cell antigen [55, 88, 89] or for divergent antibody titers resulting from natural infection with *Leucocytozoon caulleryi* [90]. Similarly, negative phenotypic correlations were identified between body weight and titers of natural antibodies (NAb) that bind keyhole limpet hemocyanin [91], a result that led the authors to postulate the existence of a genetic trade-off between levels of immunity and some production traits. Interestingly, a different result was obtained from a differential selection experiment on natural antibodies in layers conducted at Wageningen University, in which genetic and phenotypic correlations between NAb titers and body weight at different ages were, overall, very low and mostly not significant. Although a positive genetic correlation was observed between NAb levels and feed conversion ratio (FCR), the phenotypic correlation between NAb titers and FCR was very low. The observed genetic and phenotypic relationships between NAb titers and FCR led those authors to suggest that trade-offs between levels of immunity and some production traits may exist, but these are most likely not direct, with other factors, such as the digestibility of feed, playing a role [92].

The ND3 and control lines did not differ in terms of RFI. This finding indicates that the fraction of feed intake that is surplus to production and maintenance requirements is similar between lines. However, this result does not yield information about the investment in maintenance versus gain, a relationship that seems to be altered in the ND3 line because those birds were lighter than the controls. The fact that the ND3 line is lighter is a common correlated selection response in experiments for increased antibody production, as discussed above. Consequently, it is likely that variation in body weight and variation in immune response are associated to some extent. By construction, RFI is estimated after variation due to body weight (and implicitly any associated variation, such as variation in immune response) has been statistically eliminated. Therefore, it is not surprising that RFI may not discriminate between control and ND3 lines.

The underlying mechanisms of trade-offs between immunity and growth or between maintenance versus gain remain unclear, since there have only been a few studies that have investigated this subject. On the one hand, Klasing and Austic [93, 94] reported that an inflammatory challenge tends to decrease rates of protein synthesis and to increase rates of protein degradation in skeletal muscles. On the other hand, Mashaly et al. [95] found that selection for high antibody production did not cause differences in energy partitioning, but these authors did observe an increase in the amount of energy required for maintenance, which could explain the negative correlation between body weight and antibody production.

## Conclusions

This study demonstrates the usefulness of these experimental chicken lines, which have been divergently selected for RFI or for a high antibody production, as valuable models for investigations of the modulation of immune parameters in relation to the allocation of energy and nutrients within an organism. Our results are consistent with the idea that strong selection aimed at improving one specific trait may unfavorably affect other traits as a result of trade-offs between important biological functions. Of course, the correlations observed in this study between feed efficiency, growth, and immune traits are phenotypic, and further studies will be required to characterize the underlying genetic correlations between/among them. This will ultimately allow us to develop multi-trait selection programs that fully consider potential trade-offs, thus improving efficiency in livestock production without compromising immunocompetence.

## Supplementary Information

**Additional file 1: Figure S1.** RFI divergence between female and male R+ and R− chickens over 40 generations.

**Additional file 2: Table S1.** Humoral antibody response after vaccination in the R+ and R− chicken lines.

**Additional file 3: Table S2.** Whole blood leucocyte counts (× 103 cells) in vaccinated ND3 and CTR chicken lines.

## Data Availability

The datasets used in the study are available from the corresponding author on request.
