# Supplementary material for: Assessment of trade-offs between feed efficiency, growth-related traits, and immune activity in experimental lines of layer chickens
Source: Genet Sel Evol. 2021 May 6;53:44. doi: 10.1186/s12711-021-00636-z (PMC8101249; doi:10.1186/s12711-021-00636-z)
Supplement: Supplementary file 1 — Additional file 1: Figure S1. RFI divergence between female and male R+ and R− chickens over 40 generations. [file 12711_2021_636_MOESM1_ESM.docx]

Fig. S1:

RFI divergence between female and male R+ and R- chickens over 40 generations


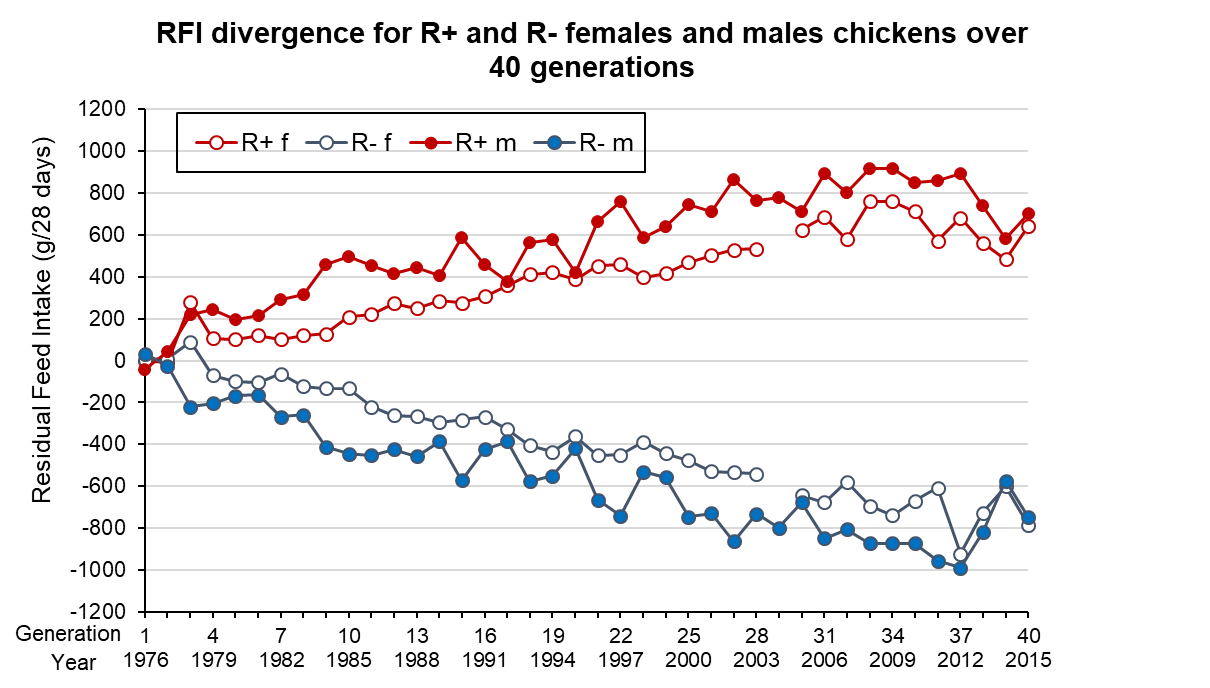


The R+ line is represented in red and the R- line in blue. Each dot is the value of residual feed intake (RFI) estimated for males and females at each generation of selection. R+ f = females from the R+ line; R- f = females from the R- line; R+ m = males from the R+ line; R- m = males from the R- line
