# Supplementary material for: Assessment of trade-offs between feed efficiency, growth-related traits, and immune activity in experimental lines of layer chickens
Source: Genet Sel Evol. 2021 May 6;53:44. doi: 10.1186/s12711-021-00636-z (PMC8101249; doi:10.1186/s12711-021-00636-z)
Supplement: Supplementary file 2 — Additional file 2: Table S1. Humoral antibody response after vaccination in the R+ and R− chicken lines. [file 12711_2021_636_MOESM2_ESM.docx]

**Additional Table 1:**

**Humoral antibody response after vaccination in the R+ and R- chicken lines**

|  | **IBV** | | **IBDV** | | | **NDV** | | **CIAV** |
| --- | --- | --- | --- | --- | --- | --- | --- | --- |
| **Weeks** | **8 wks** | **12 wks** | **8 wks** | **12 wks** | **8 wks** | | **12 wks** | **12 wks** |
| **Line^1^** |  |  |  |  |  | |  |  |
| R+ | 781 ±65 | 1287 ±152 | 4323 ±150 | 4165 ±143 | 6.3 ±0.4 | | 4.9 ±04 | 56 ±3 |
| R- | 210 ±56 | 441 ±129 | 3973 ±124 | 4166 ±122 | 3.5 ±0.3 | | 3.0 ±0.3 | 27 ±3 |
| **Sex^1^** |  |  |  |  |  | |  |  |
| Male | 491 ±77 | 647 ±170 | 4216 ±158 | 4218 ±160 | 4.7 ±0.4 | | 3.5 ±0.4 | 39.8 ±3.5 |
| Female | 498 ±74 | 1093 ±156 | 4074 ±145 | 4121 ±147 | 5.1 ±0.4 | | 4.3 ±0.3 | 43.2 ±3.2 |
| **p-value^2^** | |  |  |  |  | |  |  |
| Line | *** | *** | 0.1 | 0.9 | *** | | *** | *** |
| Sex | 0.9 | 0.2 | 0.5 | 0.7 | 0.6 | | 0.08 | 0.5 |
| **Covariate^3^** |  |  |  |  |  | |  |  |
| AWG | -1.1 ±2.8 | 3.1 ±3.0 | -2.2 ±5.2 | -2.9 ±2.8 | 0.008 ±0.02 | | 0.002 ±0.006 | 0.03 ±0.06 |
| **p-value^2^** | 0.1 | 0.1 | 0.7 | 0.3 | 0.6 | | 0.7 | 0.6 |
| **Conditional R-Squared** | 0.6 | 0.4 | 0.2 | 0.03 | 0.5 | | 0.6 | 0.5 |

Titers of vaccine-specific antibodies in sera were measured by IHA test (NDV) or by ELISA tests, either direct (IBV, IBDV, MPV) or competitive (CIAV), at 8 and 12 weeks of age. ^1^Values are least square means of antibody titers (±SE). ^2^Wald chi-square test was significant at p < 0.05*; p < 0.01**; p < 0.001***. ^3^Values are regression coefficients (± SE).
