# Supplementary material for: Assessment of trade-offs between feed efficiency, growth-related traits, and immune activity in experimental lines of layer chickens
Source: Genet Sel Evol. 2021 May 6;53:44. doi: 10.1186/s12711-021-00636-z (PMC8101249; doi:10.1186/s12711-021-00636-z)
Supplement: Supplementary file 3 — Additional file 3: Table S2. Whole blood leucocyte counts (× 103 cells) in vaccinated ND3 and CTR chicken lines. [file 12711_2021_636_MOESM3_ESM.docx]

**Table S2:**

**Whole blood leucocyte counts (x 10^3^ cells) in vaccinated ND3 and CTR chicken lines**

|  | **Heterophils** | **Monocytes** | **T cells** | **B cells** | **CD4^+^**  **helper**  **T cells** | **CD8α^+^**  **T cells** | **CD8α^+^ γδ^-^ cytotoxic T cells** | **CD8α^+^ γδ^+^**  **T cells** | **γδ^+^**  **T cells** |
| --- | --- | --- | --- | --- | --- | --- | --- | --- | --- |
| **Line^1^** |  |  |  |  |  |  |  |  |  |
| ND3 | 3.5 ±0.4 | 1.9 ±0.2 | 15.2 ±0.6 | 3.9 ±0.4 | 9.8 ±0.4 | 5.0 ±0.4 | 3.6 ±0.4 | 0. 6 ±0.1 | 6.1 ±0.3 |
| CTR | 6.5±0.4 | 2.8 ±0.2 | 20.7 ±0.6 | 5.2 ±0.4 | 10.4 ±0.4 | 7.1 ±0.4 | 5.1 ±0.3 | 1.1 ±0.1 | 8.1 ±0.3 |
| **p-value^2^** | |  |  |  |  |  |  |  |  |
| Line | *** | *** | *** | ** | 0.4 | ** | ** | ** | *** |
| **Covariate^3^** |  |  |  |  |  |  |  |  |  |
| AWG | -56.3  ±33.5 | 8.7  ±11.6 | -106.1 ±55.1 | 12.9 ±32.8 | -18.7  ±37.3 | -54.5 ±44.2 | -36.4  ±34.5 | -16.3  ±9.7 | -46.5 ±32.2 |
| **p-value^2^** | 0.1 | 0.5 | 0.1 | 0.7 | 0.6 | 0.2 | 0.3 | 0.1 | 0.1 |
| **Conditional R-Squared** | 0.6 | 0.7 | 0.4 | 0.5 | 0.02 | 0.3 | 0.4 | 0.2 | 0.2 |

^1^Values are least square means of cell counts per µl of whole blood (±SE). ^2^Wald chi-square test was significant at p < 0.05*; p < 0.01**; p < 0.001***. ^3^Values are regression coefficients (±SE)
